# SCPLPA: An miRNA–disease association prediction model based on spatial consistency projection and label propagation algorithm

**DOI:** 10.1111/jcmm.18345

**Published:** 2024-05-02

**Authors:** Min Chen, Yingwei Deng, Zejun Li, Yifan Ye, Lijun Zeng, Ziyi He, Guofang Peng

**Affiliations:** ^1^ Hunan Institute of Technology School of Computer Science and Engineering Hengyang 421002 China

**Keywords:** label propagation, miRNA–disease association prediction, spatial consistency projection

## Abstract

Identifying the association between miRNA and diseases is helpful for disease prevention, diagnosis and treatment. It is of great significance to use computational methods to predict potential human miRNA disease associations. Considering the shortcomings of existing computational methods, such as low prediction accuracy and weak generalization, we propose a new method called SCPLPA to predict miRNA–disease associations. First, a heterogeneous disease similarity network was constructed using the disease semantic similarity network and the disease Gaussian interaction spectrum kernel similarity network, while a heterogeneous miRNA similarity network was constructed using the miRNA functional similarity network and the miRNA Gaussian interaction spectrum kernel similarity network. Then, the estimated miRNA–disease association scores were evaluated by integrating the outcomes obtained by implementing label propagation algorithms in the heterogeneous disease similarity network and the heterogeneous miRNA similarity network. Finally, the spatial consistency projection algorithm of the network was used to extract miRNA disease association features to predict unverified associations between miRNA and diseases. SCPLPA was compared with four classical methods (MDHGI, NSEMDA, RFMDA and SNMFMDA), and the results of multiple evaluation metrics showed that SCPLPA exhibited the most outstanding predictive performance. Case studies have shown that SCPLPA can effectively identify miRNAs associated with colon neoplasms and kidney neoplasms. In summary, our proposed SCPLPA algorithm is easy to implement and can effectively predict miRNA disease associations, making it a reliable auxiliary tool for biomedical research.

## 1 INTRODUCTION

MicroRNAs (miRNAs), with a length of approximately 20–25 nucleotides, are a class of non‐coding RNAs that do not participate in protein coding,^1^, ^2^, ^3^ tissue differentiation,^4^ cell proliferation^2^, ^3^ and cell apoptosis.^4^, ^5^, ^6^, ^7^ However, miR‐30a‐5p, miR‐30d‐5p and miR‐30c‐5p are known to contribute to atherosclerosis and ischemic events, which are related to the development of type 2 diabetes.^8^ Currently, the understanding of miRNAs is still in its infancy, and the known functions of miRNAs represent only a small fraction. Therefore, identifying miRNAs associated with diseases will help understand the regulatory mechanisms of miRNAs and the mechanisms underlying diseases or tumour development. This work has great significance for human disease prevention and treatment.

In the wake of the discovery of a large number of miRNAs, various databases have been developed to store relevant information about miRNAs. An increasing number of bioinformatics computational methods have been developed to predict associations between miRNAs and diseases and provide assistance for further biological experimental validation. Existing prediction methods can be divided into network, machine learning and matrix factorization‐based methods.

Network‐based methods mainly aim to construct relationship networks between miRNA and diseases, proteins, environmental factors, etc. Starting from the general hypothesis in biology that ‘functionally similar miRNAs are more likely to be associated with phenotypically similar diseases, and vice versa’, corresponding algorithms are designed based on the topological structure of a relationship network. In 2009, Jiang et al.^9^ first proposed a computational model based on hypergeometric distribution to predict miRNA–disease associations. They used the relationships between miRNA‐regulated target genes to construct an miRNA functional similarity network. Xuan et al.^10^ and Chen et al.^11^ predicted unknown miRNA–disease associations by using the K‐nearest neighbour algorithm, but the accuracy of these algorithms needs to be improved. Considering that global network similarity can improve prediction accuracy more effectively than local network similarity, Chen et al.^12^ proposed a method called NetCBI, which uses network consistency to predict associations between miRNAs and diseases. Chen et al. also proposed a series of miRNA–disease association methods^13^, ^14^, ^15^ by calculating graph Laplacian scores to obtain network consistency similarity. In 2012, Chen et al.^16^ proposed a random walk‐based association prediction model called RWRMDA, which is simple to implement but cannot predict isolated diseases or new miRNAs without any known associations. Several random walk algorithms, such as MIDP,^17^ NDBM,^18^ Mugunga's method,^19^ GSTRW^20^ and NPRWR,^21^ have also been developed and achieved good prediction results. Zhan et al. proposed a model called NDALMA^22^ based on network distance analysis for predicting lncRNA–miRNA associations, and achieved good predictive performance. However, these algorithms heavily rely on known miRNA–disease (lncRNA–miRNA) associations.

Machine learning‐based methods mainly aim to use classification algorithms, such as support vector machines, decision trees, random forests and naive Bayes classifiers, especially popular deep learning methods^23^ for lncRNA–disease association and miRNA–disease association prediction. For example, Jiang et al.^24^ and Xu et al.^25^ achieved good results in using support vector machines for prediction, but the prediction performance of these models is limited by the classifiers used, such as support vector machines and decision trees. Deep learning has also been applied to this field. Zhang et al.^26^ Ji et al.^27^ Sujamol et al.^28^ and Peng et al.^29^ applied deep autoencoders to predict miRNA–disease associations. Tang et al.^30^ Dong et al.^31^ Xuan et al.^32^ Sun et al.^33^ and Wang et al.^34^ respectively applied multi‐layer convolutional neural networks for predicting miRNA–disease, metabolite–disease and lncRNA–miRNA associations. Additionally, the graph attention mechanism^35^, ^36^ has also been used in the association prediction field. These algorithms have been applied and achieved certain results in this field. However, these models still require positive and negative samples during training and have not solved the problem of selecting negative samples.

Matrix factorization‐based methods have also attracted researchers' attention. In 2017, Li et al.^37^ used matrix completion algorithms to construct an MCMDA model for prediction of miRNA–disease associations. Chen et al. improved MCMDA and developed models such as IMCMDA^38^ and NCMCMDA.^39^ Many researchers have combined matrix factorization algorithms with other methods for prediction; in particular, the NIMCGCN^40^ model combines matrix completion algorithms with graph convolutional networks, the NIMGSA^41^ model combines graph autoencoders with self‐attention mechanisms and the MDA‐AENMF^42^ model combines a five‐layer autoencoder. These models can solve the sparsity problem of heterogeneous biological data networks, but they have not effectively addressed the parameter selection problem. Additionally, many scholars have conducted extensive research in related fields,^43^, ^44^, ^45^, ^46^, ^47^, ^48^ which is also of reference value.

In summary, existing prediction models can be used to predict miRNA–disease associations but still have shortcomings, such as complex algorithm design, high computational complexity and difficulty in parameter selection. Further research is thus needed in predicting miRNA–disease associations. In the present work, a novel method, namely, SCPLPA, is proposed for prediction of miRNA–disease associations and was developed starting from the perspective of the structure of heterogeneous graphs and the heterogeneity of content.

This study constructs a heterogeneous disease similarity network composed of a disease semantic similarity network and a disease Gaussian interaction spectrum kernel similarity network as well as a heterogeneous miRNA similarity network composed of an miRNA functional similarity network and an miRNA Gaussian interaction spectrum kernel similarity network. The label propagation algorithm is then implemented in both heterogeneous networks, and their results are integrated as the initial prediction scores for miRNA–disease associations. The matrices of the heterogeneous disease similarity network and the heterogeneous miRNA similarity network are projected into the initial prediction score matrix. The two spatial projection scores are integrated as the final prediction score. As a result, multiple evaluation metrics, including AUC, AUPR, ACC, MCC and F1, indicate that SCPLPA outperforms other state‐of‐the‐art methods in terms of predictive performance. In addition, SCPLPA can predict the relationships between isolated diseases and new miRNAs. The AUC values for predicting isolated diseases and new miRNAs are 0.8412 and 0.8289, respectively. Two case results further validate the ability of SCPLPA to predict unknown miRNA associations related to diseases.

## 2 MATERIALS AND METHODS

### 2.1 Human miRNA–Disease association data

The experimentally validated miRNA–disease association data are from HMDD v2.0^49^ ${\mathrm{MD}}^{n_{m}\times n_{d}}$. If there is a known association between a miRNA $m_{i}$ and a disease node $d_{j}$, it is set $\mathrm{MD}\left(i,j\right)$ to 1; otherwise, it is set to 0. The variables nm and nd represent the number of diseases and miRNAs, respectively.

### 2.2 Disease semantic similarity

Many scholars have proposed methods to measure the semantic similarity of diseases based on disease classification information described in MeSH (Medical Subject Headings).^50^ In this method, each disease d is represented as a directed acyclic graph (DAG) $\mathrm{DAG}\left(d\right)=\left(N\left(d\right),E\left(d\right)\right)$, where $N\left(d\right)$ represents the ancestor node set of disease d (including the disease d itself) and $E\left(d\right)$ represents the set of related connections. The similarity between diseases is calculated as follows:

Xuan et al.^10^ presented the contribution value of the ancestor node $d_{a}$ of disease d to the disease d as follows:

(1) $$D_{d}\left(d_{a}\right)=-\log\left(\frac{\text{the number of}\;N\left(d\right)}{\text{the number of disease}}\right)$$

Based on Equation (1), the semantic value $\mathrm{DV}\left(d\right)$ of disease d is defined as:

(2) $$\mathrm{DV}\left(d\right)=\sum_{d_{a}\in N\left(d\right)}D_{d}\left(d_{a}\right)$$

The semantic similarity between disease $d_{i}$ and $d_{j}$ is calculated using the following equation:

(3) $$\mathrm{DD}\left(i,j\right)=\frac{\sum_{d_{t}\in N\left(d_{i}\right)\cap N\left(d_{j}\right)}D_{d_{i}}\left(d_{t}\right)+D_{d_{j}}\left(d_{t}\right)}{\mathrm{DV}\left(d_{i}\right)+\mathrm{DV}\left(d_{j}\right)}$$

The data are downloaded from the literature^51^ and named as ${\mathrm{DD}}^{n_{d}\times n_{d}}$.

### 2.3 miRNA functional similarity

The functional similarity between diseases is calculated based on the semantic similarity of diseases. The specific process is described as follows.^52^

For any two miRNAs $m_{i}$ and $m_{j}$, the sets of diseases associated with them are denoted as:

$$D^{\left(m_{i}\right)}=\left\{d_{1^{'}}\text{,}d_{2^{'}}\text{,}\ldots \text{,}d_{m^{'}}\right\}={\left\{d_{i^{'}}\right\}}_{m}\subset D\;\text{and}\;D^{\left(m_{j}\right)}=\left\{d_{1^{\prime \prime }}\text{,}d_{2^{\prime \prime }}\text{,}\ldots \text{,}d_{n^{\prime \prime }}\right\}={\left\{d_{j^{\prime \prime }}\right\}}_{n}\subset D$$

For a given disease $d_{i^{'}}$ and a given set $D^{\left(m_{j}\right)}$ of diseases, the degree of association between them is calculated as:

(4) $$S\left(d_{i^{'}},D^{\left(m_{j}\right)}\right)=\max_{d_{t}\in D^{\left(m_{j}\right)}}\left(\mathrm{DD}\left(d_{i^{'}},d_{t}\right)\right)$$

$\mathrm{DD}\left(d_{i^{'}},d_{t}\right)$ represents the semantic similarity value between disease $d_{i^{'}}$ and disease $d_{t}$.

The functional similarity between any two miRNAs $m_{i}$ and $m_{j}$ is then represented as:

(5) $${\mathrm{mm}}_{\mathrm{ij}}=\frac{\sum_{d_{t}\in D^{\left(m_{i}\right)}}S\left(d_{t},D^{\left(m_{j}\right)}\right)+\sum_{d_{t}\in D^{\left(m_{j}\right)}}S\left(d_{t},D^{\left(m_{i}\right)}\right)}{m+n}$$

In the above equation, *m* and *n* refer to the number of diseases associated with miRNA $m_{i}$ and miRNA $m_{j}$, respectively.

The matrix ${\mathrm{MM}}^{n_{m}\times n_{m}}$ is used to represent the miRNA functional similarity matrix.

### 2.4 Gaussian interaction spectral kernel similarity

When measuring the similarity between diseases by using semantic similarity method, the similarity between many diseases is directly represented as 0 due to missing data. The Gaussian kernel spectral similarity^53^ is introduced to compensate for this drawback. The similarity between disease $d_{i}$ and $d_{j}$ is defined as:

(6) $$\mathrm{GD}\left(i,j\right)=\exp\left(-\gamma_{d}∥\mathrm{mp}\left(d_{i}\right)-\mathrm{mp}\left(d_{j}\right){∥}^{2}\right)$$

where $\mathrm{mp}\left(1_{i}\right)$ represents the number of miRNAs associated with disease $d_{i}$, and $\gamma_{1}$ is the width of kernel spectrum and defined as:

(7) $$\gamma_{d}=\frac{1}{\frac{1}{n_{d}}\sum_{i=1}^{n_{d}}∥\mathrm{mp}\left(d_{i}\right){∥}^{2}}$$

The Gaussian kernel spectral similarity between miRNAs is calculated using the same method:

(8) $$\mathrm{GL}\left(i,j\right)=\exp\left(-\gamma_{1}∥\mathrm{dp}\left(m_{i}\right)-\mathrm{dp}\left(m_{j}\right){∥}^{2}\right)$$

$\mathrm{dp}\left(m_{i}\right)$ indicates the number of diseases associated with miRNA $m_{i}$, and $\gamma_{d}$ is the width of the kernel spectrum and defined as follows:

(9) $$\gamma_{m}=\frac{1}{\frac{1}{n_{m}}\sum_{i=1}^{n_{1}}∥\mathrm{dp}\left(m_{i}\right){∥}^{2}}$$

### 2.5 Integration of disease similarity and miRNA similarity

The semantic similarity between diseases and the Gaussian spectral kernel similarity between diseases are used to construct the similarity between diseases by using the following formula:

(10) $${\mathrm{DD}}^{f}\left(i,j\right)=\frac{\mathrm{GD}\left(i,j\right)+\mathrm{DD}\left(i,j\right)}{2}$$

This heterogeneous disease similarity network is represented by the matrix ${\mathrm{DD}}^{f}$.

The similarity between miRNAs is constructed by integrating miRNA functional similarity and Gaussian kernel spectral similarity as follows: if the semantic similarity between miRNA $m_{i}$ and miRNA $m_{j}$ is 0, then the similarity between miRNA $m_{i}$ and miRNA $m_{j}$ is taken as the miRNA Gaussian kernel spectral similarity $m_{i}$ between miRNA $m_{j}$ and miRNA $\mathrm{GM}\left(i,j\right)$; otherwise, it is taken as the functional similarity between miRNA $m_{i}$ and miRNA MM $m_{j}$. The formula is as follows:

(11) MMfi,j=MMi,jGMi,jifMMi,j≠0otherwise

This heterogeneous miRNA similarity network is represented by the matrix ${\mathrm{MM}}^{f}$.

### 2.6 SCPLPA

**FIGURE 1:**
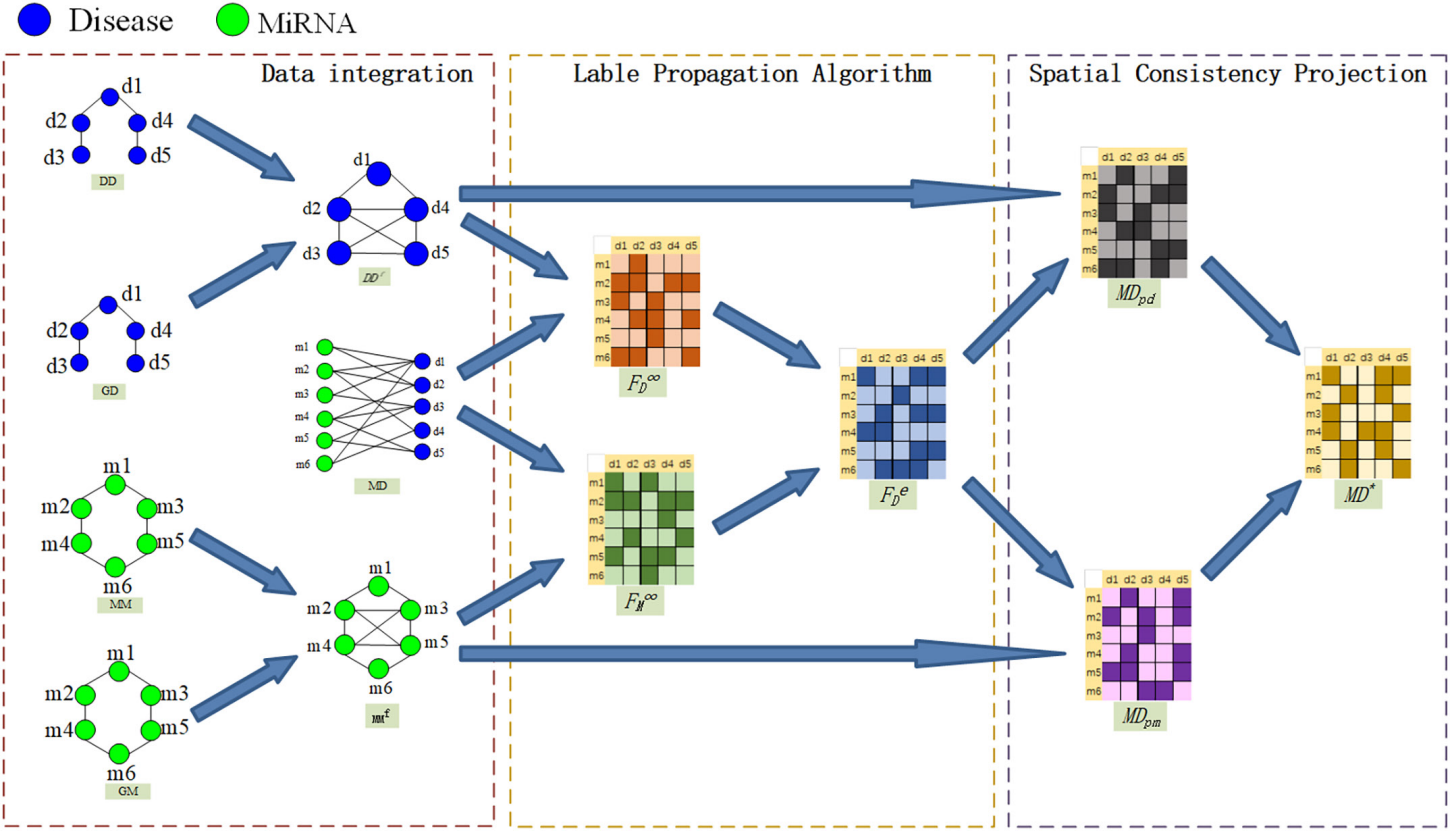
The flowchart of the whole modelling procedure.

The algorithm consists of three steps. The first step involves constructing accurate disease similarity networks and miRNA similarity networks by using heterogeneous data sources (Equations (6), (7), (8), (9), (10), (11)). The second step involves using the label propagation algorithm to obtain estimated scores for miRNA–disease associations. The third step involves using the spatial consistency projection algorithm to obtain precise scores for miRNA–disease associations. The flowchart is shown in Figure 1.

#### 2.6.1 Estimated scores for miRNA–Disease associations

The label propagation algorithm is applied separately to the heterogeneous disease similarity network and the heterogeneous miRNA similarity network to obtain initial scores for miRNA–disease associations. These initial scores are combined to obtain the estimated scores.

The label propagation algorithm in the heterogeneous disease network is defined as follows:

(12) $$F_{D}\left(t+1\right)=\left(1-\alpha \right)*{\mathrm{DD}}^{*}*F_{D}\left(t\right)+\alpha *{\mathrm{MD}}^{T}$$

In the above equation, $F_{D}\left(t\right)$ represents the t‐th iteration result of the label propagation algorithm; ${\mathrm{MD}}^{T}$ is the transpose matrix of the known miRNA–disease association matrix MD, $\alpha \in \left[0,1\right]$; and ${\mathrm{DD}}^{*}$ is the normalized matrix of the heterogeneous disease similarity network ${\mathrm{DD}}^{f}$ and is calculated as follows:

(13) $${\mathrm{DD}}^{*}\left(i,j\right)={\mathrm{DD}}^{f}\left(i,j\right)/\left(\sum_{i=1}^{\mathrm{nd}}{\mathrm{DD}}_{i,j}^{f}\left(i,j\right)+\sum_{j=1}^{\mathrm{nd}}D_{i,j}^{f}\left(i,j\right)\right)$$

$F_{D}\left(0\right)={\mathrm{MD}}^{T}$ is iterated until $\left|F_{D}\left(t+1\right)-F_{D}\left(t\right)\right|<10^{-6}$, and the iteration is then stopped. The predicted result is the initial score for miRNA–disease associations based on the heterogeneous disease similarity network, represented by the matrix $F_{D}^{\infty }$.

The label propagation algorithm in the heterogeneous miRNA network is defined by the following iteration equation:

(14) $$F_{M}\left(t+1\right)=\left(1-\beta \right)*{\mathrm{MM}}^{*}*F_{L}\left(t\right)+\beta *\mathrm{MD}$$

where $\beta \in \left[0,1\right]$, ${\mathrm{MM}}^{*}$ is the normalized matrix of the heterogeneous miRNA similarity network ${\mathrm{MM}}^{f}$ and is calculated as follows:

(15) $${\mathrm{MM}}^{*}\left(i,j\right)={\mathrm{MM}}^{f}\left(i,j\right)/\left(\sum_{i=1}^{\mathrm{nm}}{\mathrm{MM}}_{i,j}^{f}\left(i,j\right)+\sum_{j=1}^{\mathrm{nm}}{\mathrm{MM}}_{i,j}^{f}\left(i,j\right)\right)$$

$F_{M}\left(0\right)=\mathrm{MD}$ is iterated until $\left|F_{M}\left(t+1\right)-F_{M}\left(t\right)\right|<10^{-6}$, and the iteration is then terminated. The probability space reaches a stable state and is denoted as $F_{M}^{\infty }$. This value is the initial score for miRNA–disease associations based on the heterogeneous miRNA similarity network.

The predicted results $F_{L}^{\infty }$ and $F_{D}^{\infty }$ are integrated as the estimated score for miRNA–disease associations by implementing the label propagation algorithm in the two networks:

(16) $$F^{e}=\left(1-\delta \right)*F_{D}^{\infty }+\delta *F_{M}^{\infty }$$

#### 2.6.2 Accurate scores for miRNA–Disease Associations

In this stage, the spatial consistency projection algorithm is used to calculate the final predicted scores. The spatial consistency projection prediction based on the miRNA network refers to the following: in the integrated miRNA similarity matrices, if some miRNAs are highly similar to miRNA $m_{i}$ and other miRNAs highly similar to miRNAs $m_{i}$ are highly associated with disease $d_{j}$, then the credibility of the association between miRNA $m_{i}$ and disease $d_{j}$ obtains a high score. The weight of the association between miRNA $m_{i}$ and disease $d_{j}$ is calculated, and the estimated association information between miRNA $m_{i}$ and disease $d_{j}$ is obtained in the previous stage; the estimated association information between disease $d_{j}$ and all miRNAs $m_{k}=\left(k=1,2,\ldots ,\mathrm{nm}\right)$ is also utilized and combined with the similarity between miRNA $m_{i}$ and other miRNAs to calculate the credibility score between each miRNA $m_{i}$ and disease $d_{j}$. The formula is as follows:

(17) $${\mathrm{MD}}_{\mathrm{pm}}\left(i,j\right)=\frac{{\mathrm{MM}}^{f}\left(i,:\right)\times F^{e}\left(:,j\right)}{\left\|F^{e}\left(:,j\right)\right\|}$$

In the above formula, $\left\|F^{e}\left(:,j\right)\right\|$ is the 2‐norm of $F^{e}\left(:,j\right)$.

A similar approach is used to calculate the predicted scores of the spatial consistency projection based on the disease network:

(18) $${\mathrm{MD}}_{\mathrm{pd}}\left(i,j\right)=\frac{{\mathrm{DD}}^{f}\left(j,:\right)\times {\left(F^{e}\right)}^{T}\left(:,i\right)}{\left\|{\left(F^{e}\right)}^{T}(:,i)\right\|}$$

Finally, ${\mathrm{MD}}_{\mathrm{pm}}$ and ${\mathrm{MD}}_{\mathrm{pd}}$ are synthesized to obtain the final prediction score.

(19) $${\mathrm{MD}}^{*}=\varepsilon *{\mathrm{MD}}_{\mathrm{pm}}+\left(1-\varepsilon \right)*{\mathrm{MD}}_{\mathrm{pd}}^{T}$$

## 3 RESULTS

### 3.1 Evaluation metrics

We evaluated the performance of SCPLPA using LOOCV (leave‐one‐out cross‐validation), where each miRNA–disease association was selected as a test sample object once, with all other miRNA–disease associations used as the training set until all miRNA–disease associations were tested once. By setting different thresholds and plotting the ROC (receiver operating characteristic) curve with TPR (true positive rate or sensitivity) as the y‐axis and FPR (false positive rate or 1—Specificity) as the *x*‐axis, the AUC (area under the ROC curve) was calculated. The curve plotted with recall rate on the *x*‐axis and precision on the *y*‐axis is known as the PR (precision‐recall) curve. The area under the PR curve is referred to as the AUPR (area under the PR curve) value.

The formulas for TPR, FPR, precision and recall are as follows:

(20) $$\mathrm{TPR}=\frac{\mathrm{TP}}{\mathrm{TP}+\mathrm{FN}}$$

(21) $$\mathrm{FPR}=\frac{\mathrm{FP}}{\mathrm{FP}+\mathrm{TN}}$$

(22) $$\text{Precision}=\frac{\mathrm{TP}}{\mathrm{TP}+\mathrm{FP}}$$

(23) $$\text{Recall}=\frac{\mathrm{TP}}{\mathrm{TP}+\mathrm{FN}}$$

The TP (true positive) in the above formulas refers to the number of correctly predicted positive samples, that is the number of positive samples predicted as positive. FP (false positive) refers to the number of incorrectly predicted positive samples, that is the number of negative samples predicted as positive. TN (true negative) refers to the number of correctly predicted negative samples, that is the number of negative samples predicted as negative. FN (false negative) refers to the number of incorrectly predicted negative samples, that is the number of positive samples predicted as negative.

In addition to AUC and AUPR, we also used other metrics, including accuracy (ACC), F1‐score (F1) and Matthew's correlation coefficient (MCC), to evaluate the performance of the model. They are defined as follows:

(24) $$\mathrm{ACC}=\frac{\mathrm{TP}+\mathrm{TN}}{\mathrm{TP}+\mathrm{TN}+\mathrm{FP}+\mathrm{FN}}$$

(25) $$F1=\frac{1}{\frac{1}{\text{Precision}}+\frac{1}{\text{Recall}}}$$

(26) $$\mathrm{MCC}=\frac{\mathrm{TP}*\mathrm{TN}-\mathrm{FP}*\mathrm{FN}}{\sqrt{\left(\mathrm{TP}+\mathrm{FP}\right)\left(\mathrm{TP}+\mathrm{FN}\right)\left(\mathrm{TN}+\mathrm{FP}\right)\left(\mathrm{TN}+\mathrm{FN}\right)}}$$

### 3.2 Effect of parameter selection

In the Equations 12 and 14, α and β represent the probabilities of receiving initial label information in the label propagation algorithm for miRNA–disease associations, while 1−α and 1−β control the rate at which information from neighbours is retained. For simplicity, α and β are set to be the same size. The estimated score for miRNA–disease associations is calculated by weighting the prediction results $F_{L}^{\infty }$ and $F_{D}^{\infty }$ from the heterogeneous miRNA network and the heterogeneous disease network by using the label propagation algorithm, with δ representing the proportion of the two prediction results. The precision score for miRNA–disease associations is calculated by weighting the prediction scores based on miRNA spatial consistency projection and disease network spatial consistency projection, with ε representing the proportion of the two prediction results. This section mainly discusses the effect of these parameters on the predictive performance of SCPLPA.

**FIGURE 2:**
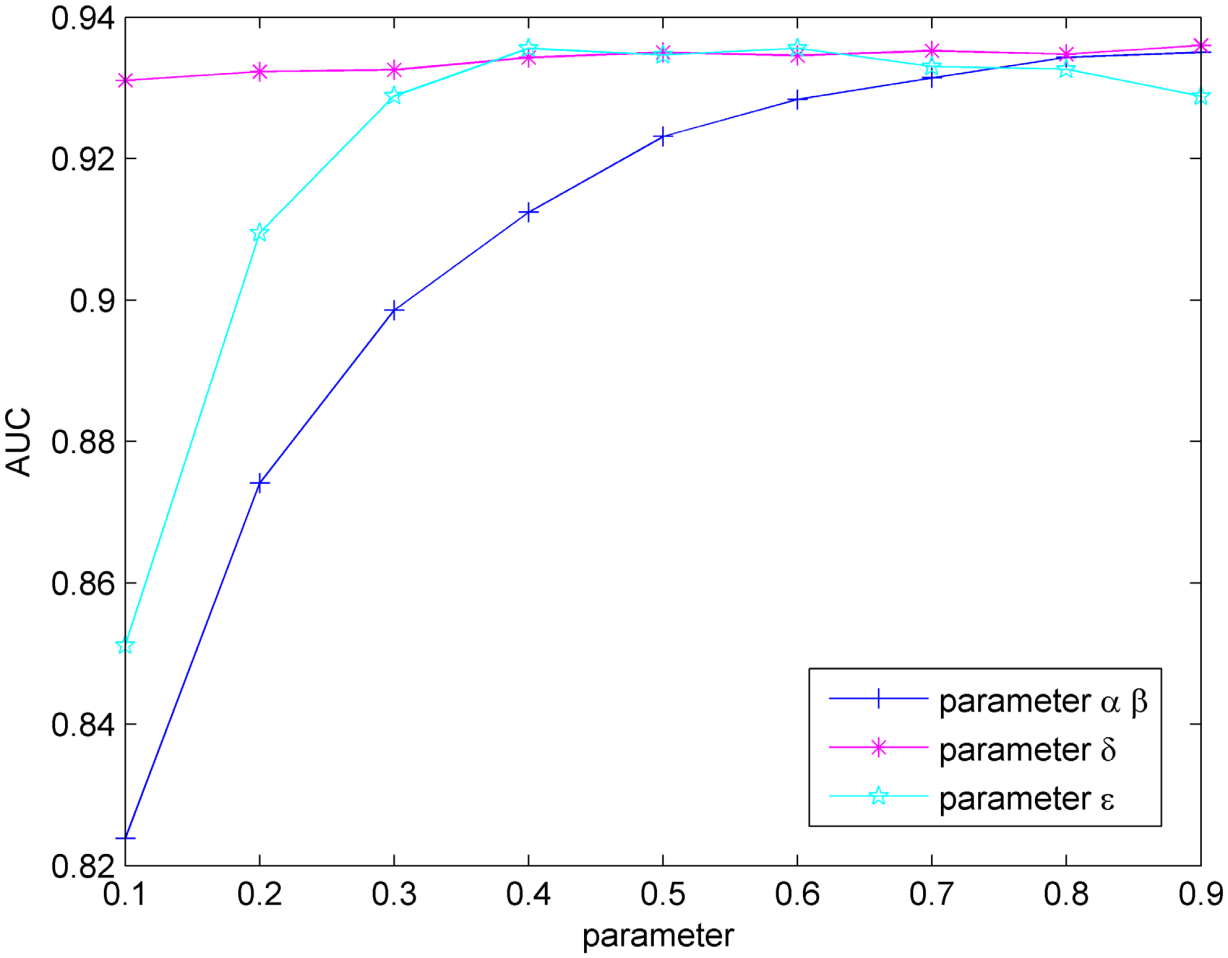
Influence of parameter variation on model predictive accuracy.

In the first step, the optimal values for α and β are determined. Here, parameters δ and ε are initially set to 0.5, with a step size of 0.1. Parameters α (or β) are increased from 0.1 to 0.9 with a step size of 0.1, and leave‐one‐out cross‐validation is performed to calculate AUC (Figure 2). When β is set to 0.9, the AUC value is maximized at 0.9335. Therefore, parameters α and β are set to 0.9. The optimal value for δ is then determined. Based on the obtained values of α = β = 0.9, the parameter ε is set to 0.5 and then the parameter δ is increased to 0.9 with a step size of 0.1. The cross‐validation is performed again to calculate the AUC values. When δ is 0.6, the AUC is maximized at 0.9346 (Figure 2). Therefore, let δ = 0.9. Finally, in the case of α = β = 0.9 and δ = 0.9, the parameter ε is increased from 0.1 to 0.9 with a step size of 0.1. When ε is 0.6, the AUC value is maximized at 0.9356. Thus, the following optimal parameter values are obtained: α = β = 0.9, δ = 0.9, ε = 0.6.

### 3.3 Comparison with state‐of‐the‐art methods

**FIGURE 3:**
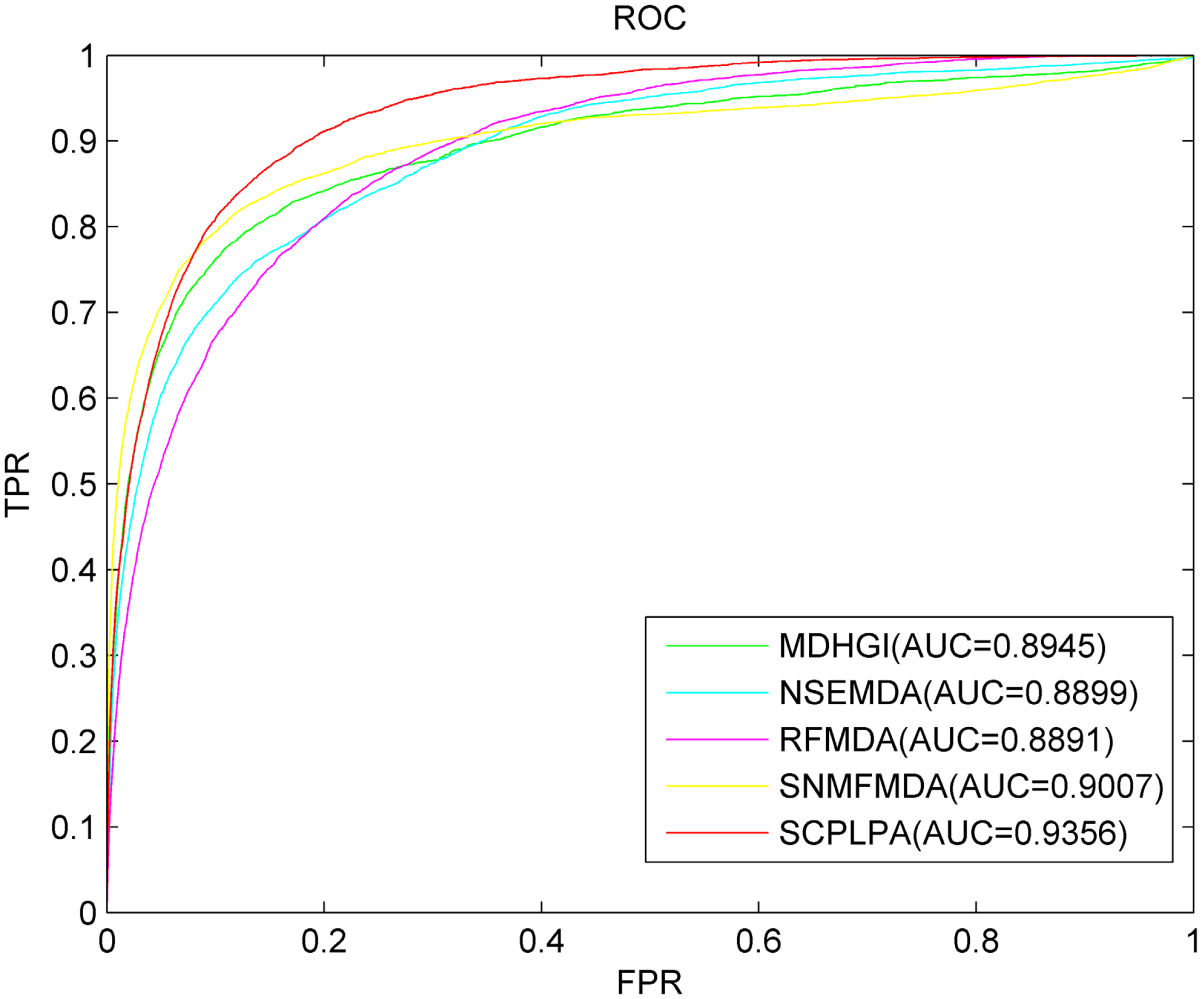
The ROC curves and AUC values of SCPLPA compared with other methods.

To the best of our knowledge, MDHGI,^54^ NSEMDA,^55^ RFMDA^56^ and SNMFMDA^57^ are excellent computational methods used to predict miRNA–disease associations. These methods utilize information similar to SCPLPA and can be used for predicting associations between isolated diseases and new miRNAs. Here, SCPLPA is compared with these methods through the parameter selection described in their respective papers. The AUC value is used as the performance metric to evaluate the prediction performance. LOOCV is performed to compare the prediction results (Figure 3). The AUC values for SCPLPA, MDHGI, NSEMDA, RFMDA and SNMFMDA are 0.9356, 0.8945, 0.8899, 0.8891 and 0.9007, respectively. To enhance the persuasiveness of our experiments, we compared SCPLPA with several other models based on AUPR, ACC, MCC and F1 values. As shown in Table 1, the AUPR value of SCPLPA is 0.4596, while MDHGI, NSEMDA, RFMDA and SNMFMDA are 0.3367, 0.3198, 0.3345 and 0.3489, respectively. SCPLPA is, respectively, higher than the other control methods by 26.74%, 30.42%, 27.22% and 24.09%. The ACC values of SCPLPA, MDHGI, NSEMDA, RFMDA and SNMFMDA are 0.5503, 0.5607, 0.5321, 0.5215 and 0.5317, respectively. SCPLPA is 1.89% lower than that of MDHGI, but respectively higher than NSEMDA, RFMDA and SNMFMDA by 3.31%, 5.23% and 3.38%.The MCC values of SCPLPA, MDHGI, NSEMDA, RFMDA and SNMFMDA are 0.1762, 0.1507, 0.1472, 0.1356 and 0.1681, respectively. SCPLPA is higher than the other comparison methods by 14.47%, 16.46%, 23.04% and 4.60%, respectively. The F1 values of SCPLPA, MDHGI, NSEMDA, RFMDA and SNMFMDA are 0.1102, 0.1023, 0.1054, 0.1012 and 0.1045, respectively. SCPLPA is higher than the other comparison methods by 7.17%, 4.36%, 8.17% and 5.17%, respectively. From these indicators, we can see that the performance of SCPLPA is significantly better than the other four methods. Overall, SCPLPA outperforms the other prediction models in terms of predictive performance.

**TABLE 1:** Comparative experimental analysis of SCPLPA and other four methods.

Method	AUC	AUPR	ACC	MCC	F1
SCPLPA	0.9356	0.4596	0.5503	0.1762	0.1102
MDHGI	0.8945	0.3367	0.5607	0.1507	0.1023
NSEMDA	0.8899	0.3198	0.5321	0.1472	0.1054
RFMDA	0.8891	0.3345	0.5215	0.1356	0.1012
SNMFMDA	0.9007	0.3489	0.5317	0.1681	0.1045

### 3.4 Prediction of new miRNAs and isolated diseases

**FIGURE 4:**
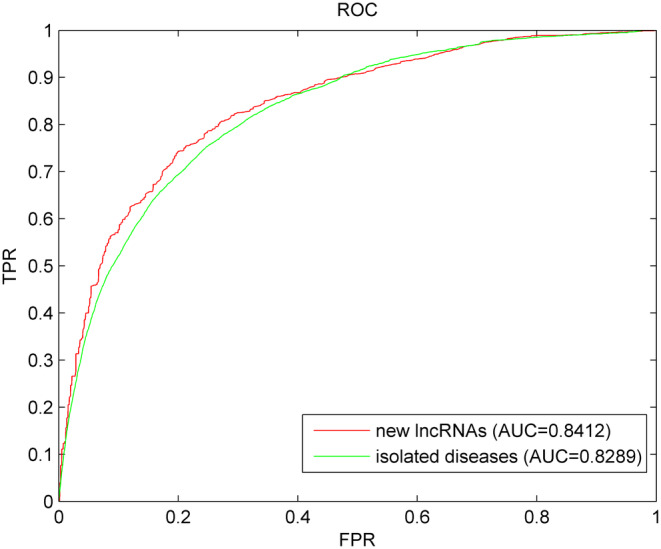
Results of SCPLPA for new miRNAs and isolated diseases.

New miRNAs have not been widely associated with specific diseases or biological functions in existing literature or databases. These miRNAs may be newly discovered, or their functions and mechanisms may not be fully understood. Rapid and accurate identification of the relationship between new miRNAs and diseases would greatly enhance our understanding of the molecular mechanisms of diseases. However, predicting the association between new miRNAs and diseases poses a significant challenge because of unknown association information. Therefore, the model cannot be directly used for prediction. The following procedure is performed once for each miRNA to further evaluate the performance of the SCPLPA model in predicting new miRNA–disease associations: first, the known associations between miRNAs to be queried and all diseases are removed, and it is simulated as a new miRNA; SCPLPA is then used for prediction. This process is repeated until each new miRNA is used as a test sample. The prediction results are evaluated using the ROC curve and AUC value. Figure 4 shows that SCPLPA achieves an AUC value of 0.8412, indicating good performance in predicting new miRNA–disease associations.

Diseases with completely unknown association information with miRNAs are named as isolated diseases. The prediction of the association between isolated diseases and miRNA is a challenging but promising research area. The association data between the disease to be predicted and all miRNAs are removed, and SCPLPA is used for prediction until each miRNA is tested once. From Figure 4, it can be seen that the AUC value is 0.8289, indicating that SCPLPA can effectively address the problem on the prediction of associations between isolated diseases and miRNAs.

### 3.5 Case analysis

Colon and kidney neoplasms were selected as case studies to demonstrate the predictive ability of the proposed SCPLPA model for disease–miRNA associations. All of the prediction results were validated in two independent databases, namely, HMDD v3.2^58^ and dbDEMC 2.0.^59^

Colon neoplasm is a tumour that poses a threat to human health and presents a complex pathological and physiological landscape.^60^ Identifying miRNAs associated with colon neoplasms plays a crucial role in understanding the pathogenesis, treatment and prognosis of these tissues. The HMDD v2.0 database contains 78 known miRNA–colon neoplasm associations, which were used as training samples to predict potential miRNAs associated with colon neoplasms. Table 2 lists the top 50 predicted miRNAs related to colon neoplasms and their supporting evidence obtained using the SCPLPA model. Among these miRNAs, 49 candidate genes were confirmed in the HMDD v3.2 and dbDEMC 2.0 databases, and only hsa‐mir‐367 was not validated. We believe that in the near future, biologists will further reveal the relationship of these miRNAs to colon neoplasms through experiments.

**TABLE 2:** The top 50 colon neoplasm‐related miRNAs.

Rank	miRNA name	Evidences	Rank	miRNA name	Evidences
1	hsa‐mir‐135a	dbDEMC	26	hsa‐mir‐34b	dbDEMC
2	hsa‐mir‐135b	HMDD, dbDEMC	27	hsa‐mir‐193a	dbDEMC
3	hsa‐mir‐18b	HMDD, dbDEMC	28	hsa‐mir‐425	dbDEMC
4	hsa‐mir‐625	dbDEMC	29	hsa‐mir‐129	dbDEMC
5	hsa‐mir‐139	dbDEMC	30	hsa‐mir‐99a	dbDEMC
6	hsa‐mir‐185	dbDEMC	31	hsa‐mir‐149	dbDEMC
7	hsa‐mir‐375	HMDD, dbDEMC	32	hsa‐mir‐34c	dbDEMC
8	hsa‐mir‐497	dbDEMC	33	hsa‐mir‐409	dbDEMC
9	hsa‐mir‐215	HMDD, dbDEMC	34	hsa‐mir‐373	dbDEMC
10	hsa‐mir‐25	HMDD, dbDEMC	35	hsa‐mir‐103a	dbDEMC
11	hsa‐mir‐27a	HMDD, dbDEMC	36	hsa‐mir‐429	HMDD, dbDEMC
12	hsa‐mir‐224	HMDD, dbDEMC	37	hsa‐mir‐124	dbDEMC
13	hsa‐mir‐302c	dbDEMC	38	hsa‐mir‐96	HMDD, dbDEMC
14	hsa‐mir‐186	dbDEMC	39	hsa‐mir‐148a	HMDD, dbDEMC
15	hsa‐mir‐338	dbDEMC	40	hsa‐mir‐339	HMDD, dbDEMC
16	hsa‐mir‐151a	dbDEMC	41	hsa‐mir‐93	HMDD, dbDEMC
17	hsa‐mir‐183	dbDEMC	42	hsa‐mir‐182	dbDEMC
18	hsa‐mir‐542	dbDEMC	43	hsa‐mir‐335	HMDD, dbDEMC
19	hsa‐mir‐345	dbDEMC	44	hsa‐mir‐320a	dbDEMC
20	hsa‐mir‐708	dbDEMC	45	hsa‐mir‐203	HMDD, dbDEMC
21	hsa‐mir‐194	HMDD, dbDEMC	46	hsa‐mir‐100	dbDEMC
22	hsa‐mir‐130a	HMDD, dbDEMC	47	hsa‐mir‐153	dbDEMC
23	hsa‐mir‐199b	dbDEMC	48	hsa‐mir‐526a	dbDEMC
24	hsa‐mir‐200a	HMDD, dbDEMC	49	hsa‐mir‐302d	dbDEMC
25	hsa‐mir‐367	Unconfirmed	50	hsa‐mir‐95	dbDEMC

Kidney neoplasm is a common tumour that has an increasing incidence rate. It has multiple histological subtypes, each has its own unique molecular characteristics. The most common subtype is clear cell renal cell carcinoma, which accounts for 75% of all cases. The 5‐year survival rate of clear cell renal cell carcinoma is less than 10%.^61^ Hence, predicting miRNAs associated with kidney neoplasms is of great practical significance.

The HMDD v2.0 database contains only seven known miRNA–kidney neoplasm‐associated pairs. These pairs were used as known information to implement SCPLPA and predict potential miRNAs associated with kidney neoplasms for the discovery of new molecular associations as prognostic or therapeutic markers. As shown in Table 3, all the top 50 predicted kidney neoplasm‐related miRNAs have been confirmed in HMDD v3.2 and dbDEMC 2.0. The two cases demonstrate that the SCPLPA model exhibits satisfactory performance in predicting new potential miRNA–disease associations.

**TABLE 3:** The top 50 kidney neoplasm‐related miRNAs.

Rank	miRNA name	Evidences	Rank	miRNA name	Evidences
1	hsa‐mir‐155	HMDD, dbDEMC	26	hsa‐mir‐134	dbDEMC
2	hsa‐mir‐146a	dbDEMC	27	hsa‐mir‐7	dbDEMC
3	hsa‐mir‐122	HMDD, dbDEMC	28	hsa‐mir‐17	HMDD, dbDEMC
4	hsa‐mir‐34a	HMDD, dbDEMC	29	hsa‐mir‐142	dbDEMC
5	hsa‐mir‐221	dbDEMC	30	hsa‐mir‐708	HMDD
6	hsa‐mir‐125b	dbDEMC	31	hsa‐mir‐9	dbDEMC
7	hsa‐mir‐16	dbDEMC	32	hsa‐mir‐184	dbDEMC
8	hsa‐mir‐29a	dbDEMC	33	hsa‐mir‐106b	dbDEMC
9	hsa‐mir‐210	HMDD, dbDEMC	34	hsa‐mir‐148a	dbDEMC
10	hsa‐mir‐31	dbDEMC	35	hsa‐mir‐19a	dbDEMC
11	hsa‐mir‐29b	dbDEMC	36	hsa‐mir‐27a	HMDD, dbDEMC
12	hsa‐mir‐199a	HMDD, dbDEMC	37	hsa‐mir‐1207	dbDEMC
13	hsa‐mir‐26a	dbDEMC	38	hsa‐mir‐19b	dbDEMC
14	hsa‐mir‐145	dbDEMC	39	hsa‐mir‐373	dbDEMC
15	hsa‐mir‐133a	dbDEMC	40	hsa‐let‐7b	dbDEMC
16	hsa‐mir‐222	dbDEMC	41	hsa‐mir‐200a	HMDD, dbDEMC
17	hsa‐mir‐196a	dbDEMC	42	hsa‐mir‐126	HMDD, dbDEMC
18	hsa‐mir‐206	dbDEMC	43	hsa‐mir‐137	dbDEMC
19	hsa‐mir‐20a	dbDEMC	44	hsa‐mir‐30b	dbDEMC
20	hsa‐mir‐1	dbDEMC	45	hsa‐mir‐34c	dbDEMC
21	hsa‐mir‐200b	dbDEMC	46	hsa‐mir‐212	dbDEMC
22	hsa‐mir‐15b	dbDEMC	47	hsa‐let‐7a	dbDEMC
23	hsa‐mir‐218	dbDEMC	48	hsa‐mir‐92a	dbDEMC
24	hsa‐mir‐29c	dbDEMC	49	hsa‐mir‐124	dbDEMC
25	hsa‐mir‐223	dbDEMC	50	hsa‐mir‐204	dbDEMC

All miRNA associations related to the disease to be validated were removed before implementing SCPLPA to test its predictive performance for isolated diseases. For colon neoplasms, 78 known colon neoplasm–miRNA associations were deleted and SCPLPA was used to predict potential miRNA–lung neoplasm associations. All the top 50 predicted miRNAs were supported by evidence in HDMM3.2 and dbDEMC databases (Table 4). Similarly, seven known kidney neoplasm–miRNA associations were deleted, and the SCPLPA model was used to predict kidney neoplasm‐related miRNAs. The top 50 predicted associations were supported by evidence in HDMM3.2 and dbDEMC (Table 5).

**TABLE 4:** The top 50 colon neoplasms‐related miRNA candidates predicted by SCPLPA with removed all known colon neoplasms‐miRNA associations and the confirmation of these associations.

Rank	miRNA name	Evidences	Rank	miRNA name	Evidences
1	hsa‐mir‐145	HMDD, dbDEMC	26	hsa‐let‐7b	HMDD, dbDEMC
2	hsa‐mir‐218	HMDD, dbDEMC	27	hsa‐mir‐101	HMDD, dbDEMC
3	hsa‐mir‐200c	HMDD, dbDEMC	28	hsa‐mir‐19a	HMDD, dbDEMC
4	hsa‐mir‐126	HMDD, dbDEMC	29	hsa‐mir‐221	HMDD, dbDEMC
5	hsa‐mir‐125b	HMDD, dbDEMC	30	hsa‐mir‐210	HMDD, dbDEMC
6	hsa‐let‐7a	HMDD, dbDEMC	31	hsa‐mir‐124	dbDEMC
7	hsa‐mir‐34a	HMDD, dbDEMC	32	hsa‐mir‐222	HMDD, dbDEMC
8	hsa‐mir‐200b	HMDD, dbDEMC	33	hsa‐mir‐148a	HMDD, dbDEMC
9	hsa‐mir‐21	HMDD, dbDEMC	34	hsa‐mir‐203	HMDD, dbDEMC
10	hsa‐mir‐16	HMDD, dbDEMC	35	hsa‐let‐7c	HMDD, dbDEMC
11	hsa‐mir‐143	HMDD, dbDEMC	36	hsa‐let‐7d	HMDD, dbDEMC
12	hsa‐mir‐31	HMDD, dbDEMC	37	hsa‐mir‐25	HMDD, dbDEMC
13	hsa‐mir‐34c	dbDEMC	38	hsa‐mir‐214	dbDEMC
14	hsa‐mir‐27a	HMDD, dbDEMC	39	hsa‐mir‐199a	dbDEMC
15	hsa‐mir‐155	HMDD, dbDEMC	40	hsa‐mir‐135a	dbDEMC
16	hsa‐mir‐183	dbDEMC	41	hsa‐mir‐181a	HMDD, dbDEMC
17	hsa‐mir‐20a	HMDD, dbDEMC	42	hsa‐mir‐196a	HMDD, dbDEMC
18	hsa‐mir‐200a	HMDD, dbDEMC	43	hsa‐mir‐18b	HMDD, dbDEMC
19	hsa‐mir‐17	HMDD, dbDEMC	44	hsa‐mir‐125a	HMDD, dbDEMC
20	hsa‐mir‐92a	HMDD, dbDEMC	45	hsa‐mir‐146b	dbDEMC
21	hsa‐mir‐34b	dbDEMC	46	hsa‐mir‐205	HMDD, dbDEMC
22	hsa‐mir‐375	HMDD, dbDEMC	47	hsa‐mir‐107	HMDD, dbDEMC
23	hsa‐mir‐182	dbDEMC	48	hsa‐mir‐142	HMDD, dbDEMC
24	hsa‐mir‐18a	HMDD, dbDEMC	49	hsa‐mir‐127	HMDD, dbDEMC
25	hsa‐mir‐10b	HMDD, dbDEMC	50	hsa‐mir‐9	dbDEMC

**TABLE 5:** The top 50 kidney neoplasms‐related miRNA candidates predicted by SCPLPA with removed all known kidney neoplasms‐miRNA associations and the confirmation of these associations.

Rank	miRNA name	evidences	Rank	miRNA name	evidences
1	hsa‐mir‐145	dbDEMC	26	hsa‐mir‐18a	dbDEMC
2	hsa‐mir‐218	dbDEMC	27	hsa‐let‐7b	dbDEMC
3	hsa‐mir‐200c	HMDD, dbDEMC	28	hsa‐mir‐10b	dbDEMC
4	hsa‐mir‐126	HMDD, dbDEMC	29	hsa‐mir‐182	dbDEMC
5	hsa‐mir‐125b	dbDEMC	30	hsa‐mir‐221	dbDEMC
6	hsa‐mir‐200b	dbDEMC	31	hsa‐mir‐210	HMDD, dbDEMC
7	hsa‐mir‐34a	HMDD, dbDEMC	32	hsa‐let‐7c	dbDEMC
8	hsa‐let‐7a	dbDEMC	33	hsa‐mir‐203	HMDD, dbDEMC
9	hsa‐mir‐21	HMDD, dbDEMC	34	hsa‐mir‐375	dbDEMC
10	hsa‐mir‐34c	dbDEMC	35	hsa‐mir‐127	dbDEMC
11	hsa‐mir‐200a	HMDD, dbDEMC	36	hsa‐mir‐9	dbDEMC
12	hsa‐mir‐143	dbDEMC	37	hsa‐mir‐124	dbDEMC
13	hsa‐mir‐20a	dbDEMC	38	hsa‐let‐7f	dbDEMC
14	hsa‐mir‐27a	HMDD, dbDEMC	39	hsa‐mir‐199a	HMDD, dbDEMC
15	hsa‐mir‐92a	dbDEMC	40	hsa‐let‐7i	dbDEMC
16	hsa‐mir‐155	HMDD, dbDEMC	41	hsa‐mir‐222	dbDEMC
17	hsa‐mir‐16	dbDEMC	42	hsa‐mir‐19b	dbDEMC
18	hsa‐mir‐101	dbDEMC	43	hsa‐mir‐100	dbDEMC
19	hsa‐mir‐17	HMDD, dbDEMC	44	hsa‐mir‐142	dbDEMC
20	hsa‐mir‐31	dbDEMC	45	hsa‐mir‐214	HMDD, dbDEMC
21	hsa‐let‐7d	dbDEMC	46	hsa‐mir‐146b	dbDEMC
22	hsa‐mir‐183	HMDD, dbDEMC	47	hsa‐mir‐223	dbDEMC
23	hsa‐mir‐34b	dbDEMC	48	hsa‐mir‐125a	dbDEMC
24	hsa‐mir‐19a	dbDEMC	49	hsa‐mir‐148a	dbDEMC
25	hsa‐mir‐205	dbDEMC	50	hsa‐mir‐146a	dbDEMC

The above experimental results further demonstrate the reliability of SCPLPA in predicting miRNAs related to isolated diseases. The model also addresses the limitation of many current miRNA–disease association prediction models in predicting miRNAs related to isolated diseases.

## 4 DISCUSSION

The association between miRNAs and diseases has attracted research attention. Variations and dysregulation of miRNAs can lead to various diseases. As such, identifying and predicting the association between miRNAs and diseases is beneficial for understanding the function and pathogenesis of miRNAs. Existing biological experimental methods for identifying miRNA–disease associations are time consuming and labour intensive. Computational prediction methods can serve as effective supplementary tools for experimental validation. Predicting potential miRNA–disease associations through computational methods has become a hot topic in bioinformatics, resulting in the development of related prediction models. However, future works should address few issues, such as low prediction accuracy, difficulty in obtaining negative samples and challenges in predicting associations for isolated diseases and new miRNAs.

This paper proposes an SCPLPA model based on network consistency projection and a label propagation algorithm to predict potential miRNA–disease associations. SCPLPA not only performs well in predicting unknown miRNA–disease interactions but also effectively predicts isolated diseases and new miRNAs.SCPLPA was compared with four state‐of‐the‐art models, namely, MDHGI, NSEMDA, RFMDA and SNMFMDA, to evaluate its performance. The ACC value of SCPLPA is 0.5503, while MDHGI, NSEMDA, RFMDA and SNMFMDA are 0.5607, 0.5321, 0.5215 and 0.5317, respectively. The AUC values of the five models obtained through LOOCV are 0.9356, 0.8945, 0.8899, 0.8891 and 0.9007, respectively. Furthermore, the AUPR value of SCPLPA is 0.4596, while MDHGI, NSEMDA, RFMDA and SNMFMDA are 0.3367, 0.3198, 0.3345 and 0.3489, respectively. SCPLPA's AUPR outperforms various state‐of‐the‐art models by at least 24.09%. This indicates that in the given datasets with imbalanced positive and negative samples, SCPLPA's predictive performance has a clear advantage over other state‐of‐the‐art models, demonstrating better robustness in handling imbalanced datasets. Additionally, the MCC values of SCPLPA, MDHGI, NSEMDA, RFMDA and SNMFMDA are 0.1762, 0.1507, 0.1472, 0.1356 and 0.1681, respectively. The F1 values of SCPLPA, MDHGI, NSEMDA, RFMDA and SNMFMDA are 0.1102, 0.1023, 0.1054, 0.1012 and 0.1045, respectively. SCPLPA also has a slight lead in F1 and MCC values. In conclusion, compared to the other four state‐of‐the‐art models, SCPLPA can improve robustness in imbalanced datasets while maintaining high prediction accuracy, showing superior performance in miRNA–disease association tasks. Each disease (miRNA) was simulated as an isolated disease (new miRNA) to evaluate the prediction performance of SCPLPA for new miRNAs and isolated diseases. Cross‐validation was then performed for each disease (miRNA). The AUC values of SCPLPA are 0.8289 (0.8412). Colon and kidney neoplasms were selected for case analysis to further validate the reliability of the SCPLPA model in predicting the relationship between potential miRNAs and diseases. In the top 50 rankings and the corresponding disease‐related miRNA predictions, the accuracy levels verified by the HDMM3.2 and dbDEMC databases are 98% and 100%, respectively. In the prediction of the isolated disease cases, all the top 50 rankings were confirmed by the two databases. The reliable predictions of SCPLPA provide insights for the identification of potential miRNA biomarkers and contribute to future research on the involvement of miRNAs in human disease mechanisms.

The outstanding predictive performance of SCPLPA is mainly due to two reasons. First, it integrates disease semantic similarity data and disease Gaussian interaction profile kernel similarity data to construct a heterogeneous disease similarity network. It also integrates miRNA functional similarity data and miRNA Gaussian interaction profile kernel similarity data to construct a heterogeneous miRNA similarity network, which can more accurately characterize the similarity between diseases and miRNAs. Second, the SCPLPA method combines the label propagation algorithm and network consistency projection sub‐models. The label propagation algorithm estimates lncRNA–disease associations, alleviates the sparsity of known miRNA–disease association data and addresses the positive and unlabelled learning problem. Consistency information between different networks is obtained, thereby solving the problems on predicting isolated diseases and new miRNAs and improving the accuracy of predicting potential miRNA–disease associations. Although SCPLPA can effectively predict miRNA–disease associations but has certain limitations. First, integrating more omics data can construct more accurate disease similarity networks and miRNA similarity networks. Second, our algorithm is based on the prediction of known miRNA–disease associations, which may lead to biased results towards diseases with known associated miRNAs. Inspired by various association prediction methods such as drug–target interaction prediction^62^ and ligand–receptor interactions,^63^, ^64^, ^65^, ^66^ we plan to explore boosting‐based or deep learning‐based models to enhance microRNA–disease prediction in future research.

## AUTHOR CONTRIBUTIONS

**Min Chen:** Conceptualization (equal); formal analysis (equal); methodology (equal); resources (equal); software (equal); supervision (equal); writing – original draft (equal); writing – review and editing (equal). **Yingwei Deng:** Conceptualization (equal); formal analysis (equal); investigation (equal); methodology (equal); resources (equal); software (equal); supervision (equal); writing – original draft (equal); writing – review and editing (equal). **Zejun Li:** Funding acquisition (equal); resources (equal). **Yifan Ye:** Investigation (equal); validation (equal); visualization (equal). **Lijun Zeng:** Project administration (equal); visualization (equal). **Ziyi He:** Investigation (equal); validation (equal); visualization (equal). **Guofang Peng:** Visualization (equal).

## CONFLICT OF INTEREST STATEMENT

The authors confirm that there are no conflicts of interest.

## Supporting information


Data S1.

Data S2.

Data S3.

Data S4.

Data S5.

## Data Availability

All datasets generated for this study are included in the article/supplementary material.

## References

[1] Mattick JS , Makunin IV . Non‐coding RNA. Hum Mol Genet. 2006;15:R17‐R29. 16651366 10.1093/hmg/ddl046

[2] Zhu L , Zhao J , Wang J , et al. MicroRNAs are involved in the regulation of ovary development in the pathogenic blood fluke *Schistosoma japonicum* . PLoS Pathog. 2016;12:e1005423. 26871705 10.1371/journal.ppat.1005423 PMC4752461

[3] Fernando TR , Rodríguez‐Malavé NI , Rao DS . MicroRNAs in B cell development and malignancy. J Hematol Oncol. 2012;5:7‐8. 22401860 10.1186/1756-8722-5-7 PMC3338094

[4] Miska EA . How microRNAs control cell division, differentiation and death. Curr Opin Genet Dev. 2005;15:563‐568. 16099643 10.1016/j.gde.2005.08.005

[5] Cheng AM , Byrom MW , Shelton J , Ford LP . Antisense inhibition of human miRNAs and indications for an involvement of miRNA in cell growth and apoptosis. Nucleic Acids Res. 2005;33:1290‐1297. 15741182 10.1093/nar/gki200 PMC552951

[6] Ambros V . MicroRNA pathways in flies and worms: growth, death, fat, stress, and timing. Cell. 2003;113:673‐676. 12809598 10.1016/s0092-8674(03)00428-8

[7] Bartel DP . MicroRNAs: target recognition and regulatory functions. Cell. 2009;136:215‐233. 19167326 10.1016/j.cell.2009.01.002 PMC3794896

[8] Pordzik J , Jakubik D , Jarosz‐Popek J , et al. Significance of circulating microRNAs in diabetes mellitus type 2 and platelet reactivity: bioinformatic analysis and review. Cardiovasc Diabetol. 2019;18:113‐131. 31470851 10.1186/s12933-019-0918-x PMC6716825

[9] Jiang Q , Hao Y , Wang G , et al. Prioritization of disease microRNAs through a human phenome‐microRNAome network. BMC Syst Biol. 2010;4:S2. 10.1186/1752-0509-4-S1-S2 PMC2880408 20522252

[10] Xuan P , Han K , Guo M , et al. Prediction of microRNAs associated with human diseases based on weighted k most similar neighbors. PLoS One. 2013;8:e70204. 23950912 10.1371/journal.pone.0070204 PMC3738541

[11] Chen X , Wu QF , Yan GY . RKNNMDA: ranking‐based KNN for MiRNA‐disease association prediction. RNA Biol. 2017;14:952‐962. 28421868 10.1080/15476286.2017.1312226 PMC5546566

[12] Chen H , Zhang Z . Similarity‐based methods for potential human microRNA‐disease association prediction. BMC Med Genet. 2013;6:12. 10.1186/1755-8794-6-12 PMC3629999 23570623

[13] Chen M , Lu X , Liao B , Li Z , Cai L , Gu C . Uncover miRNA‐disease association by exploiting global network similarity. PLoS One. 2016;11:e0166509. 27907011 10.1371/journal.pone.0166509 PMC5132253

[14] Chen M , Peng Y , Li A , et al. A novel information diffusion method based on network consistency for identifying disease related microRNAs. RSC Adv. 2018;8:36675‐36690. 35558942 10.1039/c8ra07519k PMC9088870

[15] Zhang Y , Chen M , Cheng X , Chen Z . LSGSP: a novel miRNA–disease association prediction model using a Laplacian score of the graphs and space projection federated method. RSC Adv. 2019;9:29747‐29759. 35531537 10.1039/c9ra05554a PMC9071959

[16] Chen X , Liu MX , Yan GY . RWRMDA: predicting novel human microRNA–disease associations. Mol BioSyst. 2012;8:2792‐2798. 22875290 10.1039/c2mb25180a

[17] Xuan P , Han K , Guo Y , et al. Prediction of potential disease‐associated microRNAs based on random walk. Bioinformatics. 2015;31:1805‐1815. 25618864 10.1093/bioinformatics/btv039

[18] Liao B , Ding S , Chen H , Li Z , Cai L . Identifying human microRNA–disease associations by a new diffusion‐based method. J Bioinforma Comput Biol. 2015;13:1550014. 10.1142/S0219720015500146 26004789

[19] Mugunga I , Ju Y , Liu X , Huang X . Computational prediction of human disease‐related microRNAs by path‐based random walk. Oncotarget. 2017;8:58526‐58535. 28938576 10.18632/oncotarget.17226 PMC5601672

[20] Chen M , Liao B , Li Z . Global similarity method based on a two‐tier random walk for the prediction of microRNA–disease association. Sci Rep. 2018;8:6481. 29691434 10.1038/s41598-018-24532-7 PMC5915491

[21] Li A , Deng Y , Tan Y , Chen M . A novel miRNA‐disease association prediction model using dual random walk with restart and space projection federated method. PLoS One. 2021;16(6):e0252971. 34138933 10.1371/journal.pone.0252971 PMC8211179

[22] Zhang L , Yang P , Feng H , Zhao Q , Liu H . Using network distance analysis to predict lncRNA–miRNA interactions. Interdiscip Sci. 2021;13:535‐545. 34232474 10.1007/s12539-021-00458-z

[23] Peng L , He X , Peng X , Li Z , Zhang L . STGNNks: identifying cell types in spatial transcriptomics data based on graph neural network, denoising auto‐encoder, and k‐sums clustering. Comput Biol Med. 2023;166:107440. 37738898 10.1016/j.compbiomed.2023.107440

[24] Jiang Q , Wang G , Zhang T , Wang Y . Predicting human microRNA‐disease associations based on support vector machine. *2010 IEEE International Conference on Bioinformatics and Biomedicine (BIBM)* (pp. 467–472). 2010. 10.1504/ijdmb.2013.056078 24417022

[25] Xu J , Li C‐X , Lv J‐Y , et al. Prioritizing candidate disease miRNAs by topological features in the miRNA target–dysregulated network: case study of prostate cancer. Mol Cancer Ther. 2011;10:1857‐1866. 21768329 10.1158/1535-7163.MCT-11-0055

[26] Zhang L , Chen X , Yin J . Prediction of potential miRNA–disease associations through a novel unsupervised deep learning framework with variational autoencoder. Cells. 2019;8:1040. 31489920 10.3390/cells8091040 PMC6770222

[27] Ji C , Wang Y , Gao Z , Li L , Ni J , Zheng C . A semi‐supervised learning method for MiRNA‐disease association prediction based on variational autoencoder. IEEE/ACM Trans Comput Biol Bioinform. 2022;19:2049‐2059. 33735084 10.1109/TCBB.2021.3067338

[28] Sujamol S , Vimina E , Krishnakumar U . Improving miRNA disease association prediction accuracy using integrated similarity information and deep autoencoders. IEEE/ACM Trans Comput Biol Bioinform. 2022;20:1125‐1136. 10.1109/TCBB.2022.3195514 35914051

[29] Peng J , Hui W , Li Q , et al. A learning‐based framework for miRNA‐disease association identification using neural networks. Bioinformatics. 2019;35:4364‐4371. 30977780 10.1093/bioinformatics/btz254

[30] Tang X , Luo J , Shen C , Lai Z . Multi‐view multichannel attention graph convolutional network for miRNA–disease association prediction. Brief Bioinform. 2021;22:bbab174. 33963829 10.1093/bib/bbab174

[31] Dong TN , Mucke S , Khosla M . MuCoMiD: a multitask graph convolutional learning framework for miRNA‐disease association prediction. IEEE/ACM Trans Comput Biol Bioinform. 2021;19:3081‐3092. 10.1109/TCBB.2022.3176456 35594217

[32] Xuan P , Wang D , Cui H , Zhang T , Nakaguchi T . Integration of pairwise neighbor topologies and miRNA family and cluster attributes for miRNA–disease association prediction. Brief Bioinform. 2022;23:bbab428. 34634106 10.1093/bib/bbab428

[33] Sun F , Sun J , Zhao Q . A deep learning method for predicting metabolite–disease associations via graph neural network. Brief Bioinform. 2022;23:bbac266. 35817399 10.1093/bib/bbac266

[34] Wang W , Zhang L , Sun J , Zhao Q , Shuai J . Predicting the potential human lncRNA–miRNA interactions based on graph convolution network with conditional random field. Brief Bioinform. 2022;23:bbac463. 36305458 10.1093/bib/bbac463

[35] Wang T , Sun J , Zhao Q . Investigating cardiotoxicity related with hERG channel blockers using molecular fingerprints and graph attention mechanism. Comput Biol Med. 2022;153:106464. 36584603 10.1016/j.compbiomed.2022.106464

[36] Chen Z , Zhang L , Sun J , Meng R , Yin S , Zhao Q . DCAMCP: a deep learning model based on capsule network and attention mechanism for molecular carcinogenicity prediction. J Cell Mol Med. 2023;27:3117‐3126. 37525507 10.1111/jcmm.17889 PMC10568665

[37] Li JQ , Rong ZH , Chen X , Yan GY , You ZH . MCMDA: matrix completion for MiRNA‐disease association prediction. Oncotarget. 2017;8:21187‐21199. 28177900 10.18632/oncotarget.15061 PMC5400576

[38] Chen X , Wang L , Qu J , Guan N‐N , Li J . Predicting miRNA‐disease association based on inductive matrix completion. Bioinformatics. 2018;34:4256‐4265. 29939227 10.1093/bioinformatics/bty503

[39] Chen X , Sun LG , Zhao Y . NCMCMDA: miRNA‐disease association prediction through neighborhood constraint matrix completion. Brief Bioinform. 2021;22:485‐496. 31927572 10.1093/bib/bbz159

[40] Li J , Zhang S , Liu T , Ning C , Zhang Z , Zhou W . Neural inductive matrix completion with graph convolutional networks for miRNA‐disease association prediction. Bioinformatics. 2020;36:2538‐2546. 31904845 10.1093/bioinformatics/btz965

[41] Jin C , Shi Z , Lin K , Zhang H . Predicting miRNA‐disease association based on neural inductive matrix completion with graph autoencoders and self‐attention mechanism. Biomol Ther. 2022;12:64. 10.3390/biom12010064 PMC8774034 35053212

[42] Gao H , Sun J , Wang Y , et al. Predicting metabolite–disease associations based on auto‐encoder and non‐negative matrix factorization. Brief Bioinform. 2023;24:bbad259. 37466194 10.1093/bib/bbad259

[43] Li X , Zhang P , Yin Z . Caspase‐1 and Gasdermin D afford the optimal targets with distinct switching strategies in NLRP1b inflammasome‐induced cell death. Research (Wash D C). 2022;2022:9838341. 35958114 10.34133/2022/9838341 PMC9343085

[44] Jin J , Xu F , Liu Z , Shuai J , Li X . Quantifying the underlying landscape, entropy production and biological path of the cell fate decision between apoptosis and pyroptosis. Chaos, Solitons Fractals. 2024;178:114328.

[45] Jin J , Xu F , Liu Z , et al. Biphasic amplitude oscillator characterized by distinct dynamics of trough and crest. Phys Rev E. 2023;108(6–1):064412. 38243441 10.1103/PhysRevE.108.064412

[46] Hu H , Feng Z , Lin H , et al. Modeling and analyzing single‐cell multimodal data with deep parametric inference. Brief Bioinform. 2023;24:bbad005. 36642414 10.1093/bib/bbad005

[47] Hu H , Feng Z , Lin H , et al. Gene function and cell surface protein association analysis based on single‐cell multiomics data. Comput Biol Med. 2023;157:106733. 36924730 10.1016/j.compbiomed.2023.106733

[48] Meng R , Yin S , Sun J , Hu H , Zhao Q . scAAGA: single cell data analysis framework using asymmetric autoencoder with gene attention. Comput Biol Med. 2023;165:107414. 37660567 10.1016/j.compbiomed.2023.107414

[49] Li Y , Qiu C , Tu J , et al. HMDD v2.0: a database for experimentally supported human microRNA and disease associations. Nucleic Acids Res. 2014;42:D1070‐D1074. 24194601 10.1093/nar/gkt1023 PMC3964961

[50] Lowe HJ , Barnett GO . Understanding and using the medical subject headings (MeSH) vocabulary to perform literature searches. JAMA. 1994;271:1103‐1108. 8151853

[51] Peng L‐H , Zhou L‐Q , Chen X , Piao X . A computational study of potential miRNA‐disease association inference based on ensemble learning and kernel ridge regression. Front Bioeng Biotechnol. 2020;8:40. 32117922 10.3389/fbioe.2020.00040 PMC7015868

[52] Wang D , Wang J , Lu M , Song F , Cui Q . Inferring the human microRNA functional similarity and functional network based on microRNA‐associated diseases. Bioinformatics. 2010;26:1644‐1650. 20439255 10.1093/bioinformatics/btq241

[53] van Laarhoven T , Nabuurs SB , Marchiori E . Gaussian interaction profile kernels for predicting drug–target interaction. Bioinformatics. 2011;27:3036‐3043. 21893517 10.1093/bioinformatics/btr500

[54] Chen X , Yin J , Qu J , Huang L . MDHGI: matrix decomposition and heterogeneous graph inference for miRNA‐disease association prediction. PLoS Comput Biol. 2018;14:e1006418. 30142158 10.1371/journal.pcbi.1006418 PMC6126877

[55] Wang CC , Chen X , Yin J , Qu J . An integrated framework for the identification of potential miRNA‐disease association based on novel negative samples extraction strategy. RNA Biol. 2019;16:257‐269. 30646823 10.1080/15476286.2019.1568820 PMC6380288

[56] Chen X , Wang CC , Yin J , You ZH . Novel human miRNA‐disease association inference based on random forest. Mol Ther Nucleic Acids. 2018;13:568‐579. 30439645 10.1016/j.omtn.2018.10.005 PMC6234518

[57] Zhao Y , Chen X , Yin J . A novel computational method for the identification of potential miRNA‐disease association based on symmetric non‐negative matrix factorization and Kronecker regularized least square. Front Genet. 2018;9:324. 30186308 10.3389/fgene.2018.00324 PMC6111239

[58] Huang Z , Shi J , Gao Y , et al. HMDD v3. 0: a database for experimentally supported human microRNA–disease associations. Nucleic Acids Res. 2018;47:D1013‐D1017. 10.1093/nar/gky1010 PMC6323994 30364956

[59] Yang Z , Wu L , Wang A , et al. dbDEMC 2.0: updated database of differentially expressed miRNAs in human cancers. Nucleic Acids Res. 2017;45:D812‐D818. 27899556 10.1093/nar/gkw1079 PMC5210560

[60] Madeo G , Bonetti G , Gadler M , et al. Omics sciences and precision medicine in colon cancer. Clin Ter. 2023;174:55‐67. 37994749 10.7417/CT.2023.2472

[61] Turajlic S , Swanton C , Boshoff C . Kidney cancer: the next decade. J Exp Med. 2018;215:2477‐2479. 30217855 10.1084/jem.20181617 PMC6170181

[62] Peng L , Liu X , Yang L , et al. BINDTI: a bi‐directional intention network for drug‐target interaction identification based on attention mechanisms. IEEE J Biomed Health Inform. 2024;11. doi: 10.1109/JBHI.2024.3375025 38457318

[63] Peng L , Tan J , Xiong W , et al. Deciphering ligand–receptor‐mediated intercellular communication based on ensemble deep learning and the joint scoring strategy from single‐cell transcriptomic data. Comput Biol Med. 2023;163:107137. 37364528 10.1016/j.compbiomed.2023.107137

[64] Peng L , Xiong W , Han C , Li Z , Chen X . CellDialog: a computational framework for ligand‐receptor‐mediated cell‐cell communication analysis. IEEE J Biomed Health Informatics. 2024;28:580‐591. 10.1109/JBHI.2023.3333828 37976192

[65] Peng L , Yuan R , Han C , et al. CellEnBoost: a boosting‐based ligand‐receptor interaction identification model for cell‐to‐cell communication inference. IEEE Trans Nanobioscience. 2023;22:705‐715. 37216267 10.1109/TNB.2023.3278685

[66] Peng L , Gao P , Xiong W , Li Z , Chen X . Identifying potential ligand–receptor interactions based on gradient boosted neural network and interpretable boosting machine for intercellular communication analysis. Comput Biol Med. 2024;171:108110. 38367445 10.1016/j.compbiomed.2024.108110

