# Supplementary material for: SCPLPA: An miRNA–disease association prediction model based on spatial consistency projection and label propagation algorithm
Source: J Cell Mol Med. 2024 May 2;28(9):e18345. doi: 10.1111/jcmm.18345 (PMC11063733; doi:10.1111/jcmm.18345)
Supplement: Supplementary file 5 — Data S5. [file JCMM-28-e18345-s002.docx]

**Additional fle 1:Disease Semantic Similarity**

**Additional fle 2**:Known miRNA-disease associations.

**Additional fle 3**: diseases_list.

**Additional fle 4:**miRNAs_list.
